# The Combination of RAD001 and NVP-BEZ235 Exerts Synergistic Anticancer Activity against Non-Small Cell Lung Cancer *In Vitro* and *In Vivo*

**DOI:** 10.1371/journal.pone.0020899

**Published:** 2011-06-14

**Authors:** Cheng-Xiong Xu, Yikun Li, Ping Yue, Taofeek K. Owonikoko, Suresh S. Ramalingam, Fadlo R. Khuri, Shi-Yong Sun

**Affiliations:** Department of Hematology and Medical Oncology, Emory University School of Medicine and Winship Cancer Institute, Atlanta, Georgia, United States of America; Wayne State University, United States of America

## Abstract

The phosphoinositide 3-kinase (PI3K)-mammalian target of rapamycin (mTOR) signaling axis has emerged as a novel target for cancer therapy. Agents that inhibit PI3K, mTOR or both are currently under development. The mTOR allosteric inhibitor, RAD001, and the PI3K/mTOR dual kinase inhibitor, BEZ235, are examples of these agents. We were interested in developing strategies to enhance mTOR-targeted caner therapy. In this study, we found that BEZ235 alone effectively inhibited the growth of rapamycin-resistant cancer cells. Interestingly, the combination of sub-optimal concentrations of RAD001 and BEZ235 exerted synergistic inhibition of the growth of human lung cancer cells along with induction of apoptosis and G1 arrest. Furthermore, the combination was also more effective than either agent alone in inhibiting the growth of lung cancer xenografts in mice. The combination showed enhanced effects on inhibiting mTOR signaling and reducing the expression of c-Myc and cyclin D1. Taken together, our results suggest that the combination of RAD001 and BEZ235 is a novel strategy for cancer therapy.

## Introduction

K-Ras, LKB1 and epidermal growth factor receptor (EGFR) are frequently mutated in non-small cell lung cancer (NSCLC). These mutations result in aberrant activation of the phosphoinositide 3-kinase (PI3K)/Akt/mammalian target of rapamycin (mTOR) signaling pathway [1], [2], [3]. Therefore, the PI3K/Akt/mTOR signaling pathway has emerged as a promising therapeutic target for NSCLC.

RAD001 (Everolimus) is a derivative of rapamycin and is functionally similar to rapamycin as an allosteric inhibitor of mTOR. In patients with advanced renal cell cancer previously treated with VEGF targeted agents, RAD001 improves progression-free survival and has therefore been approved by the US Food and Drug Administration for this indication [4]. It has also been found to improve progression-free survival in patients with neuroendcorine cancers of the pancreas. In many other solid organ malignancies, RAD001 and other rapamycin analogues (rapalogs) the rapalogs exert modest anti-cancer effects, that though promising, are not sufficient to warrant monotherapy with these agents [5].

Recent efforts to improve the efficacy of the rapalogs have focused on developing novel combination strategies. NVP-BEZ235 (BEZ235) is a novel and orally administered dual PI3K and mTOR kinase inhibitor. This compound is a potent, reversible inhibitor of both class I PI3K and mTOR kinase catalytic activity by competing at their ATP-binding site [6]. BEZ235 is currently under evaluation in phase I/II clinical trials. In preclinical studies, BEZ235 induces striking anti-proliferative effects both in transgenic mice with oncogenic K-Ras-induced NSCLC and in NSCLC cell lines expressing oncogenic K-Ras. Moreover, it effectively sensitizes NSCLC cell lines expressing oncogenic K-Ras to the pro-apoptotic effects of ionizing radiation both *in vitro* and *in vivo* [7]. When BEZ235 was combined with a MEK inhibitor, marked synergy was achieved in shrinking K-Ras mutant murine lung cancers [8].

Like rapamycin, RAD001 causes Akt activation in human cancer cells including NSCLC cells while inhibiting the mTOR signaling [9]. We recently reported on the enhanced efficacy of the combination of RAD001 with a PI3K inhibitor on the growth of NSCLC cells both *in vitro* and *in vivo* [9]. Interestingly, BEZ235 could overcome rapamycin resistance as it effectively inhibited the growth of rapamycin- or RAD001-resistant NSCLC cells. Therefore we evaluated the effects of the combination of RAD001 and BEZ235 on the growth of NSCLC cells and found that the combination was more effective than either agent alone in inhibiting the growth of NSCLC cells both *in vitro* and *in vivo*. This report will primarily document our research findings in this regard.

## Materials and Methods

### Reagent

RAD001 and BEZ235 were supplied by Novartis Pharmaceuticals Corporation (East Hanover, NJ), dissolved in DMSO and stored at −80°C. Rabbit polyclonal anti-actin antibody was purchased from Sigma Chemical Co. (St. Louis, MO). Antibodies against Akt, p-Akt (S473), p-S6 (S235/S236), S6, p-4EBP1 (S65) p-4EBP1 (Thr37/46), 4EBP1, eIF4G, eIF4E, and poly(ADP-ribose)polymerase (PARP), respectively, were purchased from Cell Signaling Technology, Inc. (Beverly, MA). Goat polyclonal mTOR (FRAP; N-19) and mouse monoclonal c-Myc (9E10) antibodies were purchased from Santa Cruz Biotechnology, Inc. (Santa Cruz, CA), respectively. Rabbit polyclonal Rictor (BL2178) antibody was purchased from Bethyl Laboratories, Inc. (Montgomery, TX). Mouse monoclonal cyclin D1 antibody was purchased from Dako (Carpinteria, CA).

### Cell Lines and Cell Culture

The human NSCLC cell lines A549, H460 and H157 were described previously [10]. HCC827 was purchased from the American Type Culture Collection ATCC (Manassas, VA). Rapamycin-resistant A549 cell line (A549-RR) was established previously [9]. These cell lines were grown in monolayer culture in RPMI 1640 medium supplemented with 5% fetal bovine serum (FBS) at 37°C in a humidified atmosphere consisting of 5% CO_2_ and 95% air.

### Growth Inhibition Assay

Cells were cultured in 96-well cell culture plates and treated the next day with the agents indicated. Viable cell number was estimated using the sulforhodamine B (SRB) assay, as previously described [10]. Combination index (CI) for drug interaction (e.g., synergy) was calculated using the CompuSyn software (ComboSyn, Inc.; Paramus, NJ).

### Colony Formation Assay

The effects of the given drugs on colony formation on plates were measured as previously described [11].

### Detection of Apoptosis

Apoptosis was evaluated by Annexin V staining using Annexin V-PE apoptosis detection kit purchased from BD Biosciences (San Jose, CA) according to the manufacturer's instructions.

### Western Blot Analysis

Preparation of whole cell protein lysates and Western blot analysis were described previously [12], [13].

### m^7^GTP Pull-down for Analysis of eIF4F Complex Formation

eIF4F complex in cell extracts was detected using affinity chromatography m^7^GTP-Sepharose as described previously [14].

### Detection of mTOR complexes (mTORCs)

mTORCs including mTORC1 and mTORC2 were immunoprecipitated with goat polyclonal mTOR (FRAP; N-19) antibody and followed with Western blotting to detect mTOR, raptor and rictor, respectively, as described previously [9].

### Lung Cancer Xenografts and Treatments

Animal experiments were approved by the Institutional Animal Care and Use Committee (IACUC) of Emory University. The protocol number is 222-2008. Five- to 6-week old female athymic (nu/nu) mice were ordered from Taconic (Hudson, NY) and housed under pathogen-free conditions in microisolator cages with laboratory chow and water *ad libitum*. A549 cells at 5×10^6^ in serum-free medium were injected s.c. into the flank region of nude mice. When tumors reached a size of approximately 100 mm^3^, the mice were randomized into four groups (n = 6/group) according to tumor volumes and body weights for the following treatments: vehicle control, BEZ235 (20 mg/kg/day, og), RAD001 (3 mg/kg/day; og), and their combination. Tumor volumes were measured using caliper measurements once every two days and calculated with the formula *V* = π(length×width^2^)/6.

### Statistic Analysis

The statistical significance of differences between two groups or among multiple groups was analyzed with two-sided unpaired Student's *t* tests (for equal variances) or with Welch's corrected *t* test (unequal variances) or one-way analysis of variance (ANOVA) by use of Graphpad InStat 3 software. Results were considered to be statistically significant at *P*<0.05.

## Results

### BEZ235 Effectively Inhibits the Growth of Rapamycin-resistant NSCLC Cells

**Figure 1:**
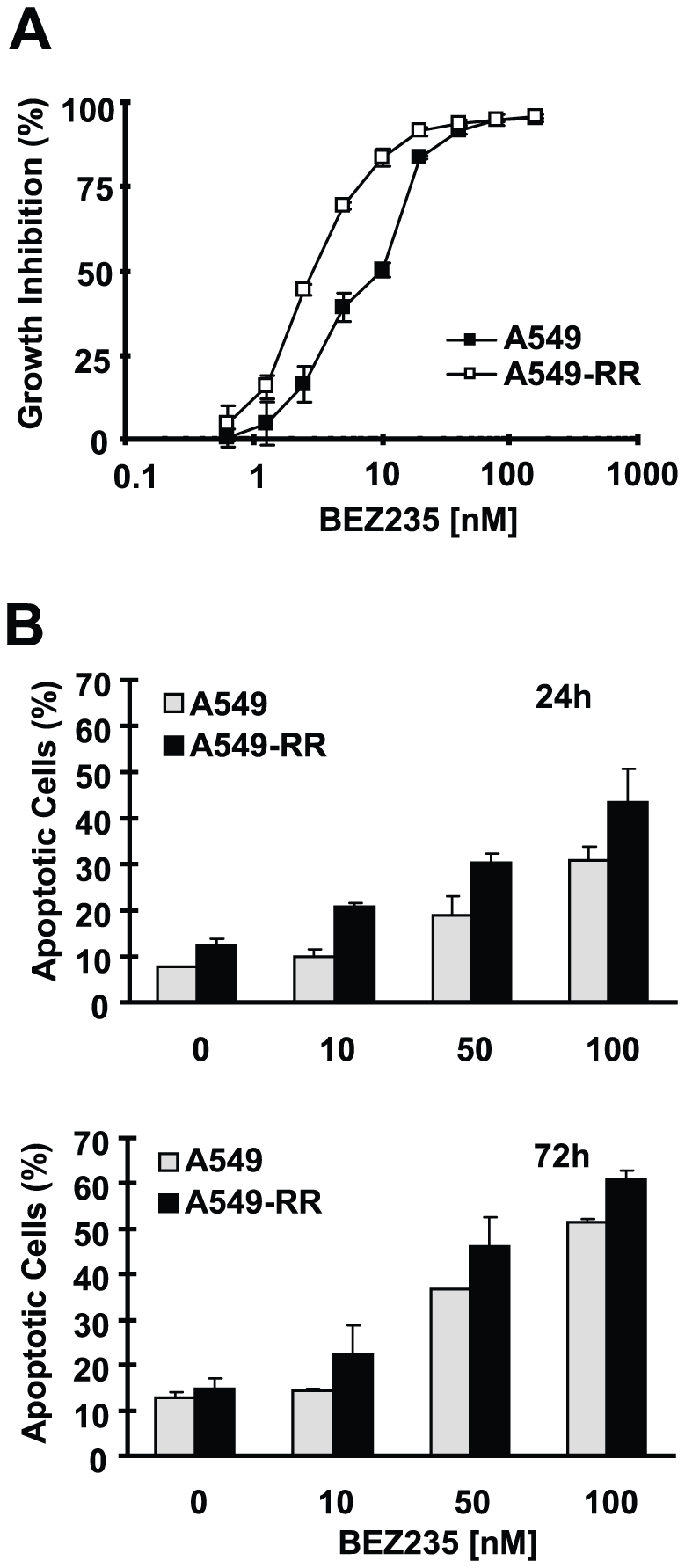
BEZ235 is effective in inhibiting the growth (*A*) and inducing apoptosis (*B*) of rapamycin-resistant cells. *A*, The indicated cell lines were seeded in 96-well plates and then treated with different concentrations of BEZ235 as indicated on the second day. After 3 days, the cell numbers were estimated using SRB assay. Points, means of four replicate determinations; bars, ± SD. *B*, The indicated cell lines were plated in 6-well plates and then treated next day with different concentrations of BEZ235 as indicated. After 24 and 72 h, the cells were harvested and subjected to detection of apoptosis using Annexin V staining. Columns, means of duplicate determinations; bars, ± SD.

In a prior study, we established a rapamycin-resistant cell line (i.e., A549-RR). This cell line is also resistant to RAD001 [9]. We anticipated that this cell line would be, at least in part, resistant to BEZ235 since it is a PI3K and mTOR dual inhibitor. Unexpectedly, BEZ235 demonstrated potent inhibition of the growth of A549-RR cells (Fig. 1A). Moreover, BEZ235 also induced apoptosis in A549-RR cells (Fig. 1B). In fact, the induction of apoptosis and growth inhibition with BEZ235 was slightly more effective in A549-RR cell than in the parent A549 cells (Fig. 1). Thus, rapamycin-resistant cells do not show cross-resistance to BEZ235.

### The Combination of RAD001 and BEZ235 Synergistically Inhibits the Growth of NSCLC Cells along with Induction of Apoptosis and G1 arrest

**Figure 2:**
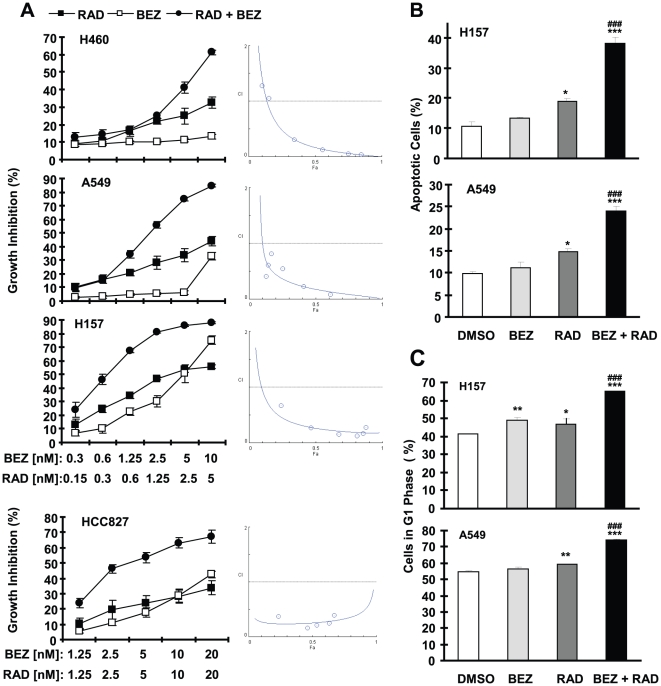
The combination of BEZ235 and RAD001 synergistically inhibits cell growth (*A*) and induces apoptosis (*B*) and cell cycle arrest (*C*) in NSCLC cells. *A*, the indicated cell lines were seeded in 96-well plates and then treated next day with different concentrations of BEZ235 (BEZ), RAD001 (RAD) and their respective combinations as indicated. After 3 days, the cell numbers were estimated using the SRB assay and CIs were calculated with CompuSyn software (right panels). Points, means of four replicate determinations; bars, ± SD. *B* and *C*, The indicated cell lines were seeded in 6-well plates and then treated with 10 nM BEZ235 alone, 2 nM RAD001 alone, and their combination. After 48 h, the cells were harvested for detection of apoptosis using Annexin V staining (*A*) and for cell cycle analysis with a flow cytometry (*C*). Columns, means of duplicate determinations; Bars, ± SD. *, *P*<0.05, ** *P*<0.01, and ***, *P*<0.001 compared with DMSO control; ###, *P*<0.001 compared with RAD001 or BEZ235 alone.

We previously demonstrated that the combination of rapamycin or RAD001 with the PI3K inhibitor LY294002 resulted in enhanced growth-inhibitory effects against NSCLC cells both *in vitro* and *in vivo* [9], [15]. We have now studied whether the combination of BEZ235 and RAD001 exerts augmented anti-cancer activity in NSCLC cells. Unexpectedly, we found that the combination of low concentrations of BEZ235 and RAD001 was much more potent than each single agent in inhibiting the growth of several NSCLC cell lines (e.g., A549, H460, H157 and HCC827). The CIs for most combinations were <1 (Fig. 2A, right panels), indicating synergistic effects on inhibiting the growth of NSCLC cells. In agreement, the combination of BEZ235 and RAD001 was significantly more potent than each single agent in inducing apoptosis (Fig. 2B) and G1 arrest (Fig. 2C) (*P*<0.001). Thus, enhanced induction of both apoptosis and cell cycle arrest contributes to augmented growth-inhibitory effects induced by the combination.

### The Combination of RAD001 and BEZ235 Effectively Inhibits the Formation and Growth of NSCLC Cell Colonies

**Figure 3:**
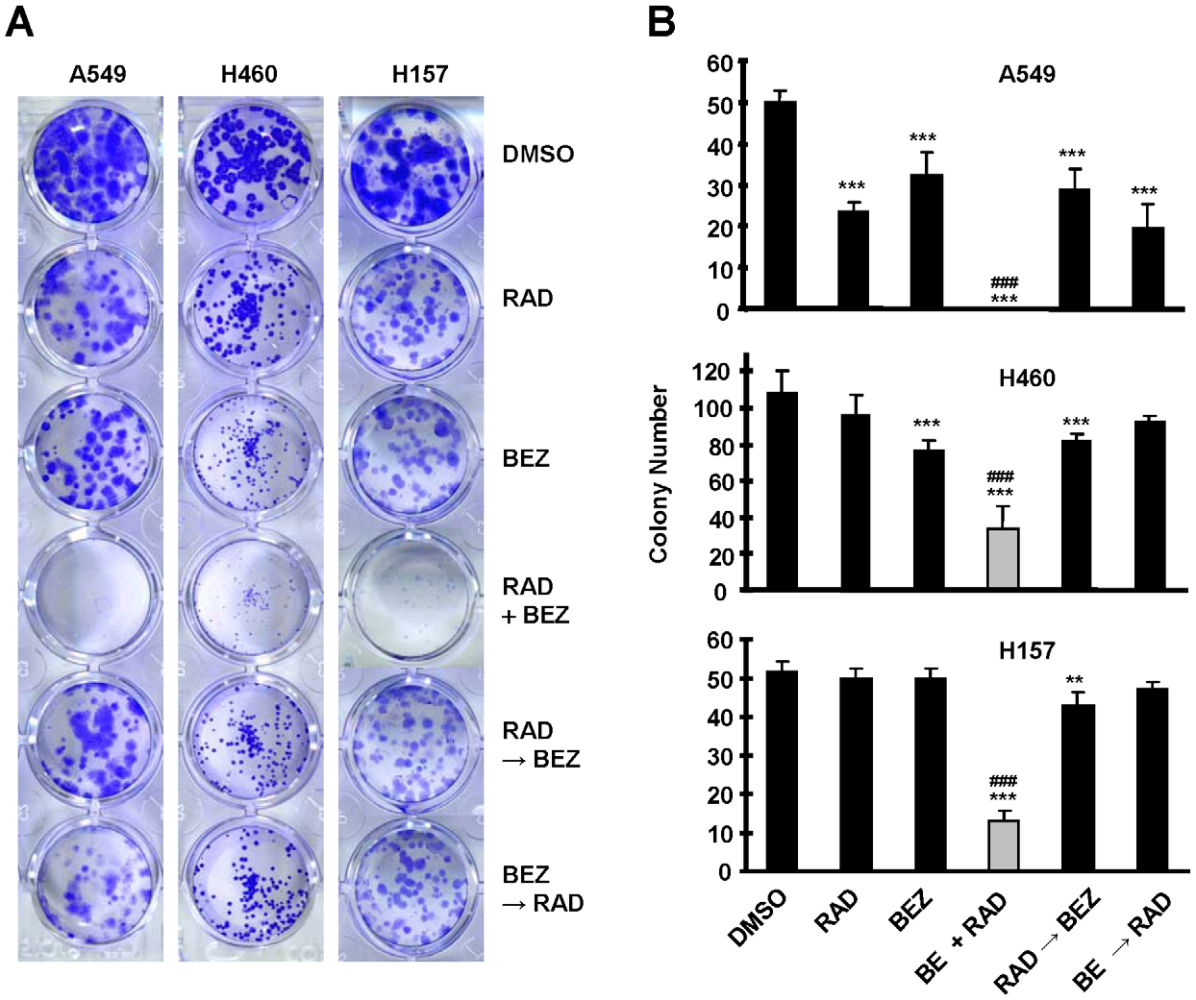
The concurrent combination of BEZ235 and RAD001 effectively inhibits colony formation ands growth of NSCLC cell. The indicated cell lines at a density of approximately 200 cells/well were seeded in 24-well plates. On the second day, the cells were treated with 1 nM RAD001 (RAD), 5 nM BEZ235 (BEZ) or their concurrent combinations (RAD+BEZ). The cells were also treated with 1 nM RAD001 for 6 days followed with 5 nM BEZ235 for another 6 days (RAD→BEZ) or with 5 nM BEZ for 6 days followed with 1 nM RAD001 for another 6 days (BEZ→RAD). After 12 days, the plates were stained for the formation of cell colonies with crystal violet dye. The picture of the colonies was then taken using a digital camera (*A*) and the colony numbers were counted (*B*). ***, *P*<0.001 compared with DMSO control; ###, *P*<0.001 compared with all other treatments.

We further determined the long-term effects of the combination of RAD001 and BEZ235 on the growth of NSCLC cells in a colony formation assay. This assay allows us to repeat the treatments for a long time (e.g., 12 days). RAD001 at a dose of 1 nM and BEZ235 at 5 nM alone had minimal effect on suppression of colony formation of the NSCLC cells; however the combination either eliminated the colony formation (e.g., A549) or drastically reduced the colony numbers (e.g., H460 and H157) (Fig. 3). Thus, it is clear that the combination is much more effective than either single agent in inhibiting the colony formation and growth of NSCLC cells (*P*<0.001). We also compared the effect of sequence of administration of the two agents on colony formation of NSCLC cells. Under the same experimental conditions described above, sequential treatments with RAD001 first followed by BEZ235 treatment (RAD001→BEZ235) or BEZ235 first followed by RAD001 treatment (BEZ235→RAD001) showed effects comparable to each alone with minimal suppression of the growth of NSCLC cell colonies. The concurrent combination of RAD001 and BEZ235 was much more potent than either sequential treatment in inhibiting the formation and growth of NSCLC colonies (*P*<0.001) (Fig. 2). Therefore, concurrent administration of RAD001 and BEZ235 is clearly superior to sequential treatments in inhibiting the growth of NSCLC cell colonies.

We further compared the effects of the combination of RAD001 and LY294002 with sequential treatments on colony formation of NSCLC cells. Consistently, the concurrent combination treatment, but not the sequential treatment either with RAD001 first followed by LY294002 or with LY294002 followed by RAD001, generated augmented effects on inhibiting the colony formation of NSCLC cells (Fig. S1).

### The Combination of RAD001 and BEZ235 Exerts Augmented Activity against the Growth of NSCLC Xenografts in Nude Mice

**Figure 4:**
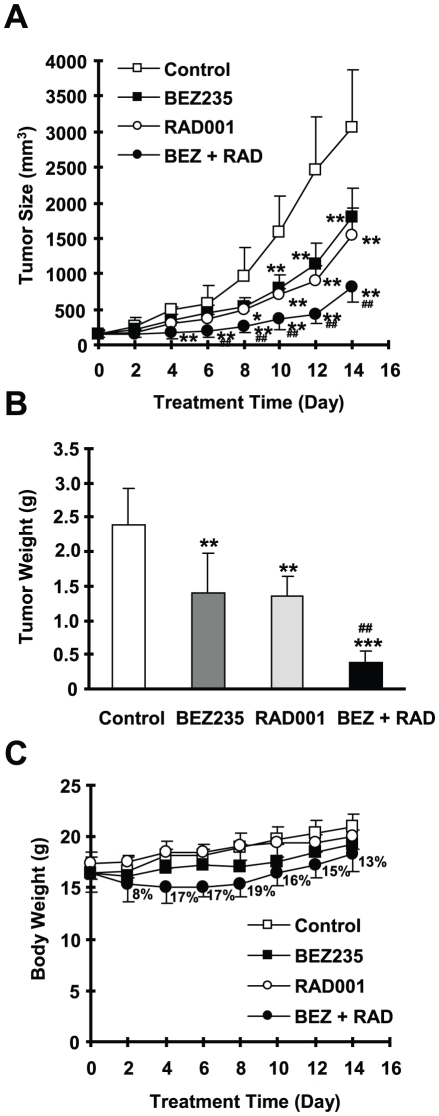
The combination of BEZ235 and RAD001 is significantly more effective than each single agent in suppressing the growth of NSCLC xenografts. A549 xenografts were treated (once a day) with vehicle control, RAD001 (3 mg/kg, og), BEZ235 (20 mg/kg, og) and their combination (BEZ+RAD) starting on the same day after grouping. Tumor sizes (*A*) and body weight (*C*) were measured once every two days. After 14 days, the mice were sacrificed and the tumors were removed and weighed (*B*). Each measurement is a mean ± SD (n = 6). The numbers in *C* represent body weight loss in the combination group compared with control group. * *P*<0.05 compared with vehicle control; ** *P*<0.01 compared with vehicle control; *** *P*<0.001 compared with vehicle control; ^##^ *P*<0.01 compared with RAD001 or with BEZ235.

Because of the promising growth-inhibitory effects of the RAD001 and BEZ235 combination in NSCLC cells *in vitro*, we then validated the efficacy of the combination against the growth of NSCLC tumors in mice. Both RAD001 and BEZ235 partially, but significantly, inhibited the growth of A549 xenografts (*P*<0.01); however the combination of RAD001 and BEZ235 was significantly more potent than each single agent in inhibiting the growth of the xenografts as measured by both tumor sizes and weights (*P*<0.01) (Figs. 4A and 4B). These *in vivo* data further demonstrate that the combination of RAD001 and BEZ235 displays augmented anticancer activity. We observed a higher degree of weight loss in mice treated with the combination (up to 19% of control mice) especially during the early treatment period. The weight difference at the end of the experiment improved to only 13% of control (Fig. 4C), suggesting possible adaptation and better tolerance of the combination treatment,

### The Combination of RAD001 and BEZ235 Exerts Enhanced Effects on Suppression of the mTOR signaling and Downregulation of c-Myc and Cyclin D1

**Figure 5:**
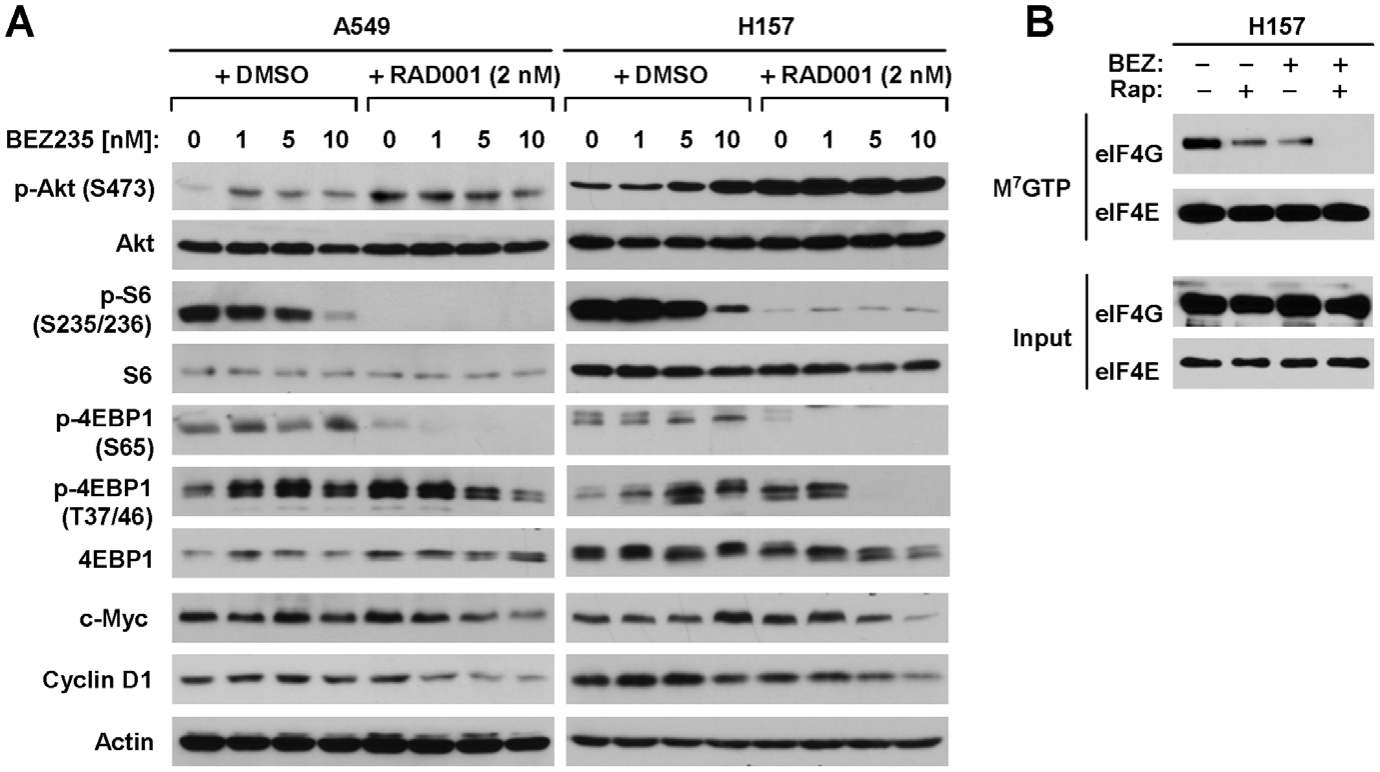
Effects of BEZ235 and RAD001 combination on the mTOR signaling (A), on the expression of mTOR-regulated proteins (A) and on the assembly of eIF4F complex (B). *A*, The indicated cell lines were plated in 10 cm-diameter cell culture dishes and treated next day with the given concentrations of BEZ-235 in the absence and presence of RAD001 for 24 h. The cells were then harvested for preparation of whole-cell protein lysates and subsequent Western blot analysis to detect the indicated proteins. *B*, The indicated cell lines were treated with 2 nM RAD001, 10 nM BEZ235 or their combination. After 24 h, the cells were harvested for preparation of whole-cell protein lysates and subsequent m^7^GTP pull-down assay followed with Western blot analysis to detect the given proteins.

To gain insight into the mechanisms by which the combination of RAD001 and BEZ235 exert enhanced anticancer activity, we analyzed the effects of the combination on mTOR signaling and on the expression of its regulated proteins in comparison with either agent alone. At the tested doses, BEZ235 had a minimal effect on reduced p-S6 levels, but no effect on the levels of p-4EBP1 (both S65 and T37/46), c-Myc and cyclin D1. In fact, we observed increased levels of 4EBP1 (T37/46) (in both A549 and H157) and c-Myc (e.g., in H157). RAD001 at 2 nM strongly inhibited S6 and 4EBP1 (S65) phosphorylation, but did not reduce the levels of p-4EBP1 (T37/46), c-Myc and Cyclin D1. Similar to BEZ235, RAD001 also increased the levels of p-4EBP1 (T37/46) and c-Myc in both A549 and H157 cells. However the combination of RAD001 and BEZ235 either abrogated the increase in p-4EBP1 (T37/46) (e.g., in A549 cells) induced by the single agent or exerted enhanced effect on reducing p-4EBP1 (T37/46) levels (e.g., in H157 cells). Importantly, the combination of RAD001 and BEZ235 had augmented effects on decreasing the levels of c-Myc and cyclin D1 in both A549 and H157 cells in comparison with each single agent alone (Fig. 5).

RAD001 increased Akt phosphorylation in both A549 and H157 cell lines as we previously reported [9]. Interestingly, at low doses, BEZ235 also increased p-Akt levels. The presence of BEZ235 at the tested dose ranges either weakly reduced the levels of p-Akt induced by RAD001 (e.g., in A549 cells) or did not affect RAD001-induced increase in p-Akt (e.g., in H157 cells) (Fig. 5). Thus, it seems that the RAD001 and BEZ235 combination can display enhanced effects on suppressing the mTOR signaling and the expression of its regulated proteins with limited or no inhibitory effects on Akt phophorylation.

### The Combination of RAD001 and BEZ235 Exerts Enhanced Effects on Suppressing eIF4F Assembly

Since mTOR signaling is known to positively regulate cap-dependent translation initiation, we further analyzed the effects of RAD001 and BEZ235 combination on the cap binding of eIF4E and eIF4G (e.g., eIF4F assembly) with the m^7^GTP-Sepharose pull down assay. As presented in Fig. 5B, RAD0001 and BEZ235 alone reduced the amounts of eIF4G that interacted with eIF4E. However, the combination of RAD001 and BEZ235 was much more effective that either agent alone in decreasing the amounts of eIF4G binding to eIF4E. Theses results clearly indicate that the combination of RAD001 and BEZ235 exerts enhanced effects on suppressing the cap binding of eIF4E and eIF4G or eIF4F assembly.

### The Combination of RAD001 and BEZ235 Does not Exhibit Enhanced Effects on Inhibiting the Assembly of mTORCs

**Figure 6:**
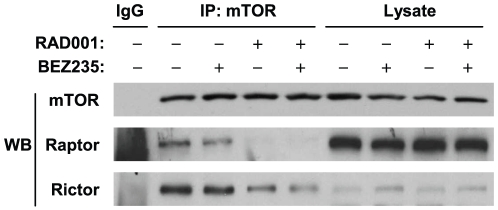
The combination of RAD001 and BEZ235 does not enhance disruption of mTORC assembly. A549 cells were treated with 2 nM RAD001, 10 nM BEZ235 or their combination. After 24 h, the cells were harvested for preparation of whole-cell protein lysates and subsequent IP-Western blotting.

It is known that the assembly or association of the mTOR with its partners (e.g., raptor and rictor) is essential for distinct enzyme activities and biological functions. RAD001, like rapamycin, suppresses mTOR signaling by inhibiting the assembly of the mTORCs [16]. Thus, we further determined whether the combination of RAD001 and BEZ235 exerted enhanced inhibitory effects on the assembly of the mTORCs including mTORC1 (mTOR/raptor) and mTORC2 (mTOR/rictor). To this end, we did immunoprecipitation (IP) with anti-mTOR antibody to pull down both mTORC1 and mTORC2 and then followed with Western blotting to detect raptor and rictor in the immunoprecipitates. As presented in Fig. 6, BEZ235 had minimal effects on reducing the levels of raptor and rictor in the immunoprecipitates, whereas RAD001 substantially reduced the levels of both raptor and rictor pulled down by mTOR antibody. The combination of RAD001 and BEZ235 had similar potency to RAD001 alone in reduction of the levels of raptor and rictor in the immunoprecipitates, indicating that the combination does not exhibit enhanced effects on inhibiting the assembly of mTORC1 and mTORC2.

## Discussion

Development of rapamycin resistance is a critical issue in the treatment of cancer with rapamycin and its analogues [17]. BEZ235 is a PI3K and mTOR dual kinase inhibitor [6]. Our study demonstrated that BEZ235 inhibited the growth of rapamycin-resistant cells and induced apoptosis as effectively as it did in the matched parent cells. In fact, rapamycin-resistant cells were slightly more sensitive than their parental cells to BEZ235 (Fig. 1). These data suggest that rapamycin-resistant cells are not cross-resistant to BEZ235. Since this cell line had been shown to be fully resistant to RAD001, our findings suggest that BEZ235 inhibits the growth of cancer cells through different mechanisms from those that mediate the actions of rapalogs. It will be interesting to know if BEZ235 possess additional mechanism beyond dual inhibition of PI3K and BEZ235. Beside, our data also imply that BEZ235 can be used to overcome rapamycin resistance.

Although BEZ235 inhibits both PI3K and mTOR, in combination with RAD001, it exerts synergistic effects in inhibiting the growth of a panel of NSCLC cells as demonstrated in a 3-day monolayer culture (with the SRB assay) and in a long-term 12 days colony formation assay (Figs. 2 and 3). This synergy is likely due to enhanced effects on induction of cell cycle G1 arrest and apoptosis (Fig. 2). In agreement, the combination of RAD001 and BEZ235 was significantly more effective than either agent in inhibiting the growth of NSCLC xenografts in nude mice (Fig. 4). In the animal study, we noted that the combination initially caused significant loss of body weight (up to 19% of control); however, at the end of the experiment, mice receiving the combination treatment seemed to recover some of the weight loss (13% of control). This suggests that the mice can adapt and eventually tolerate the treatment with the combination of RAD001 and BEZ235. Nonetheless, we should aware potential enhanced adverse effects caused by the combination while the combination shows promising synergistic anticancer activity.

Treatment schedules may impact the final outcome of the given combinational therapy. In this study, we found that the sequential treatments with RAD001 followed by BEZ235 or with BEZ235 followed by RAD001 minimally inhibited the growth of NSCLC colonies; in contrast, the concurrent treatment of RAD001 and BEZ235 substantially inhibited growth of NSCLC colonies or eliminated the colony formation (Fig. 3). This is also true for the combination of rapamycin and LY294002 (Fig. S1). Our data suggests that the concurrent combination of RAD001 and BEZ235 may be optimal for further development of this combination.

The IC_50_s (concentrations of inhibiting 50% cell growth) of BEZ235 in human NSCLC cells range from 10 nM to 100 nM (our unpublished data). In our combination experiments, we typically used low dose ranges of BEZ235 (e.g., 1–10 nM). At these doses, BEZ235 had a weak inhibitory effect on p-S6 phosphorylation but did not modulate p-4EBP1 phosphorylation or the levels of c-Myc and cyclin D1. At a dose of 2 nM, RAD001 effectively inhibited the phosphorylation of S6 and 4EBP1 (S65), but did not suppress 4EBP1 phosphorylation (T37/46) and c-Myc and cyclin D1 expression. However, the combination of RAD001 and BEZ235 effectively inhibited p-4EBP1 phosphorylation (at T37/46) and reduced the levels of c-Myc and cyclin D1 (Fig. 5A). Moreover, we showed that the combination of RAD001 and BEZ235 was much more potent than either single agent in inhibiting the cap binding of eIF4E and eIF4E or eIF4F assembly (Fig. 5B), implying that the combination exerts enhanced inhibitory effect on cap-dependent initiation. Since c-Myc and cyclin D1 are known to be regulated by the mTOR signaling through cap-dependent protein translation [18], our data indicate that the combination of RAD001 and BEZ235 exerts enhanced effect on inhibiting the mTOR signaling and the expression of its regulated oncogenic proteins (e.g., c-Myc and cyclin D1). This effect may contribute to the synergistic activity against the growth of NSCLC cells *in vitro* and *in vivo* by the combination of RAD001 and BEZ235.

In this study, RAD001 increased Akt phosphorylation in both in A549 and H157 cells; this is in agreement with our previous reports [9]. At the concentrations tested (e.g., 1–10 nM), BEZ235 increased p-Akt levels as well. This observation is consistent with a previous report, in which BEZ235 was shown to increase Akt phosphorylation at low doses (e.g., 10 nM) [19]. It had been previously shown that higher concentrations of BEZ235 are needed (e.g., >100 nM) to inhibit Akt compared with that (e.g., >10 nM) required for inhibiting S6 phosphorylation [19]. Thus, it appears that BEZ235 primarily possesses mTOR-inhibitory activity at the low concentrations ranges. Accordingly, it is understandable that BEZ235 at low concentration ranges increases Akt phosphorylation as would be expected of a rapalog [9], [15]. Interestingly, the combination of RAD001 and BEZ235 did not reduce p-Akt levels, which were as high as those in cells treated with RAD001 or BEZ235 alone (Fig. 5). Given that the combination of RAD001 and BEZ235 effectively inhibits the growth of NSCLC cells as discussed above, it appears that the combination of RAD001 and BEZ235 can exert enhanced anticancer activity with elevated levels of p-Akt.

mTOR exerts its critical roles in promoting cell cycle progression and cell proliferation primarily through interactions with other proteins such as raptor (forming mTORC1) and rictor (forming mTORC2) [18], [20]. mTORC2 is generally thought to be insensitive to rapalogs [18]. However, prolonged treatment with these mTOR inhibitors disrupts the assembly of the mTORC2 as demonstrated by us [9] and others [21]. In this study, after a 24 h treatment, RAD001, but not BEZ235, effectively inhibit the assembly or activity of both mTORC1 and mTORC2. The combination of RAD001 and BEZ235 did not further reduce the levels of raptor and rictor in the immunoprecipitates (Fig. 6), demonstrating that the combination does not display enhanced effects on inhibiting the assembly of mTORCs. Based on these observations, we speculate that the enhanced effects on suppression of the mTOR signaling by the combination is likely due to their distinctive effects on inhibiting the mTORC assembly and mTOR kinase activity. It is generally believe that a synergy is achieved through a corporation of two drugs functioning via distinct mechanisms. Since BEZ235 effectively inhibits the growth of the rapamycin-resistant cells, it is also possible that the synergy between RAD001 and BEZ235 against the growth of lung cancer cells occurs through an unknown mechanism of BEZ235, which needs further investigation.

In summary, the current study has demonstrated that the combination of RAD001 and the PI3K/mTOR inhibitor BEZ235 exhibits synergistic inhibition on the growth of NSCLC cells *in vitro* and *in vivo* and thus represents a novel strategy to enhance the efficacy of mTOR-targeted cancer therapy. Our findings provide the rationale to evaluate this combination in clinical trials for patients with rapalog-sensitive and refractory malignancies.

## Supporting Information

Figure S1 **Concurrent combination of rapamycin and LY294002 is more effective than sequential treatments in inhibiting the formation and growth of NSCLC colonies.** The indicated cell lines at a density of approximately 200 cells/well were seeded in 24-well plates. On the second day, cells were treated with 1 nM rapamycin (Rap) alone, 5 nM LY294002 (LY) alone, concurrent combination of rapamycin and LY294002 (Rap+LY), rapamycin for 3 days and then switched to LY294002 treatment (Rap→LY), LY294002 for 3 days and then switched to rapamycin treatment (LY→Rap). The same cycles of the treatments were repeated every 3 days. After 12 days, the plates were stained for the formation of cell colonies with crystal violet dye. The picture of the colonies was then taken using a digital camera (A) and the colonies were counted (*B*).
(PDF)
