# Supplementary figures and images for: The Combination of RAD001 and NVP-BEZ235 Exerts Synergistic Anticancer Activity against Non-Small Cell Lung Cancer In Vitro and In Vivo
Source: PLoS One. 2011 Jun 14;6(6):e20899. doi: 10.1371/journal.pone.0020899 (PMC3114848; doi:10.1371/journal.pone.0020899)

**A**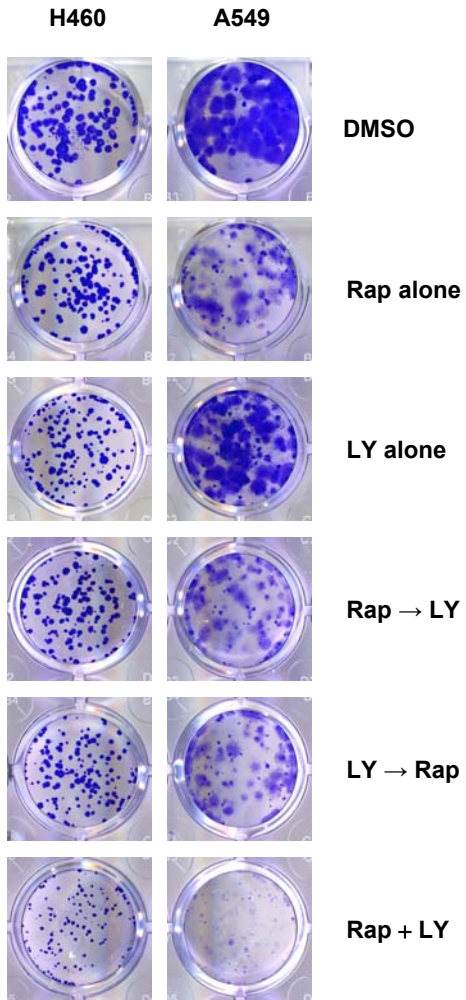**B**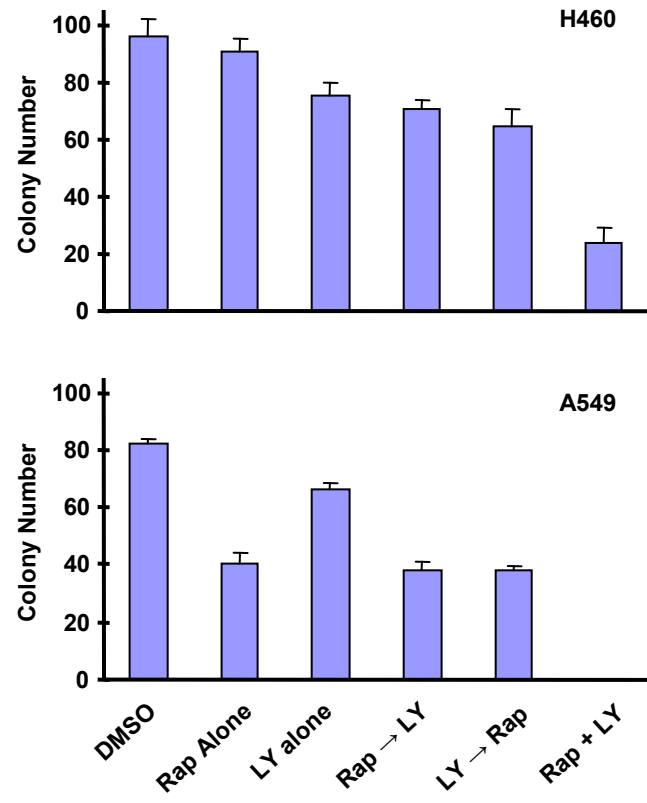

Supplement: Figure S1 — Concurrent combination of rapamycin and LY294002 is more effective than sequential treatments in inhibiting the formation and growth of NSCLC colonies. The indicated cell lines at a density of approximately 200 cells/well were seeded in 24-well plates. On the second day, cells were treated with 1 nM rapamycin (Rap) alone, 5 nM LY294002 (LY) alone, concurrent combination of rapamycin and LY294002 (Rap+LY), rapamycin for 3 days and then switched to LY294002 treatment (Rap→LY), LY294002 for 3 days and then switched to rapamycin treatment (LY→Rap). The same cycles of the treatments were repeated every 3 days. After 12 days, the plates were stained for the formation of cell colonies with crystal violet dye. The picture of the colonies was then taken using a digital camera (A) and the colonies were counted (B). (PDF) [file pone.0020899.s001.pdf]
